# Interview with Yuji Kuroiwa: a novel approach to the ageing challenge

**DOI:** 10.2471/BLT.17.031117

**Published:** 2017-11-01

## Abstract

Addressing the needs of Japan’s rapidly ageing population is a major challenge for policy-makers. Yuji Kuroiwa tells Andréia Azevedo Soares why that challenge is an opportunity to boost wellbeing and growth.

Q: You were a journalist for many years, how did you become interested in public health?

A: While working as the anchor of a national television news programme, I was shocked to learn that patients were dying due to the lack of medical services in ambulances in Japan. Between 1989 and 1991, I campaigned for ambulance teams to provide emergency medical services and did several television reports on how other countries, such as the United States of America and France, include physicians or paramedics in their ambulance teams. Soon after that, Japan developed its own system of emergency medical care. Our campaign had contributed to this and this made me realize the potential influence civil society can have in health-care decision-making.

Q: How did you become interested in issues related to older people?

A: My father was diagnosed with liver cancer and given two months to live in 2005. He received chemotherapy in hospital and suffered from the side-effects and, eventually, asked to go home and receive Kampo, a form of traditional Japanese medicine, to relieve the side-effects. He recovered from the side-effects and could take up his usual daily routine again for the last months. This experience made me acutely aware of the importance of what we call the *me-byo* approach, which emphasizes living a healthy life and preserving our quality of life for as long as possible.

Q: Why are ageing-related issues a priority for you?

A: Kanagawa has a population of 9.1 million. We are one of the fastest ageing prefectures in Japan. Currently, the proportion of people aged 65 and above in the prefecture is 24% – in Japan we refer to this age group as “older people” – and we project that by 2050, one in every 142 people will be over 100 years of age, due to increased life expectancy and declining birth rates. Also, by 2050, the over-85-years age group will be the largest group in the demographic pyramid of our prefecture. If we don’t act now, our society will become unsustainable and collapse.

Q: Did you campaign on health issues when you first ran as governor in 2011?

A: The election took place just after the Great East Japan Earthquake in March 2011 and so renewable energy was my first priority, but I also appealed to voters to create a society where people are healthy and functional into old age. Our goal is not to keep health-care costs down, but to create a society that can age, smile and go forward. This idea is at the heart of the policy package that we launched in the prefecture in 2014, called Healthcare New Frontier. However, if we implement this policy, our medical expenses should come down.

Q: Can you tell us about this policy package?

A: Our prefecture is now planning for the health and welfare needs of its citizens, assuming that they will live 100 years. Healthcare New Frontier is a bundle of health-care and industrial strategies that seek to promote the health and wellbeing of our population. These policies include the promotion of health-care information and communication technologies, and the establishment of a new department in Kanagawa's graduate school on health innovation, which will open in 2019. These policies have been defined in line with the *me-byo* concept. It’s an ancient Chinese word to which we gave a new interpretation. It refers to the gradual changes between sickness and health, rather than the duality of sickness and health. *Me-byo* is not so much about treating or curing sickness but preserving health and well-being as far as possible.

Q: Is the concept related to prevention?

A: It is broader than prevention in western medicine which is based on the idea that you are either healthy or sick. Prevention does not apply if people are sick. If you apply *me-byo*, you can improve a sick person’s condition through healthy food, daily exercise and social participation. Older people often stop going out and may become isolated due to their frailty. They need a sense of dignity and purpose, so we encourage social activities, such as workshops where they can discuss common issues and learn how to remain healthy and active for as long as possible. We also organize vocational training for older people who would like to start a new business.

“Our prefecture is now planning for the health and welfare needs of its citizens, assuming that they will live 100 years.”

Q: What are the results of your policy so far?

A: We have just started. I am a member of the Council of Health-care Policy Advisors that advises the Japanese cabinet. Following my proposal, our national health policy, which was approved by the cabinet in 2014, included a description of *me-byo* as an approach to the challenges of a rapidly ageing population. Last month we held the second Me-byo Summit to discuss the approach with stakeholders from Japan and other countries. In the Kanagawa prefecture, we are trying to integrate the concept in new technologies, such as genomics, regenerative medicine, big data solutions and robotics. The policy package aims to create stimulate economic growth with what we call the "*me-byo* industry".

*Q: What is the *me-byo* industry?*

A: The promotion of health and wellbeing is the ultimate aim of the Healthcare New Frontier policy package, but we also aim to stimulate economic growth, which benefits the population and increases tax revenues, thus increasing local government ability to provide the services that are needed. To this end, Kanagawa Prefecture registered ME-BYO™ as an international trademark in 2015. The idea is to grant the use of this trademark to trustworthy companies, so that they can display it at events organized by the Kanagawa Prefecture or others providing related services and products.

Q: What kinds of technologies do you anticipate?

A: Some technologies can be used to monitor older people’s health status. One example is a mobile phone application that analyses your voice and monitors your health. This voice analysis can help to diagnose mental disorders, heart disease and brain disease. Another example is a device that can be installed in toilets with a sensor to analyse flatulence, urine and excrement. The results can indicate, for example, whether someone needs to be tested for diabetes or colon cancer.

Q: Are these devices intended for private or nursing homes? Are they affordable?

A: Currently, we are promoting a model house with sensors installed in the toilet for private homes. This is our model for the future, especially for older people who live in remote areas and do not have easy access to health services. For example, many toilet lids in Japan open automatically when the person enters the room and the toilets flush automatically. This technology used to be expensive, now nearly every home in Japan has it. The same should happen with *me-byo* technology.

Q: What other kinds of technology are being developed?

A: Our prefecture is testing a new project called My Me-byo Record that we developed with the private sector. This is a mobile phone application that enables individuals to monitor their own health and register information, for example, about the medicines they are taking. It is a personal record the individual can use to manage his or her daily health status as opposed to the medical records kept in hospitals. The data are protected and stored in an online location. If your formal medical records are inaccessible, or destroyed in an earthquake, you may be able to retrieve your personal data.

“We are about to create a new insurance model inspired by the me-byo approach according to which the insurance company charges you less if you are making an effort to improve your health status.”

Q: Are you adapting the current health insurance model to your new approach?

A: Currently, health-care systems cover people’s treatment costs when they are sick, but there is no financial incentive to improve their health. We are about to create a new insurance model inspired by the *me-byo* approach according to which, the insurance company charges you less if you are making an effort to improve your health status. So, for example, if your personal health record shows that you have been exercising, you would be rewarded with lower premiums. This doesn’t mean that people who are not making an effort are penalized with higher premiums. Next year the health-care insurance system in Japan will change and this will provide an opportunity to create a new insurance model.

Q: Does this redesign of insurance aim to make the provision of health care more financially sustainable?

A: Our primary goal is to extend people’s healthy life expectancy, recognizing of course that this may help to reduce medical expenses in the future, because it aims to incentivize healthy behaviour and this should lead to less ill health and lower medical bills.

Q: How are you addressing the social determinants of health: the factors such as housing, employment, transport and environment that determine an individual’s health, but that may be beyond the control of the individual?

A: We will establish a new department in graduate school of Kanagawa University of Human Services in which students can study innovation in addition to public health for the purpose of solving social issues in the field of health care. We will provide this new education with future generations in mind, in collaboration with academia and research organizations in Japan and abroad.

Q: What is the public response to your policies so far?

A: While integrating the *me-byo* concept into different interventions in Kanagawa, we have been inviting suggestions from the public. We have held a series of forums, these are meetings held across the prefecture, where I had the chance to talk to members of the public and to listen to their comments and suggestions. We also receive feedback via Twitter. Our idea is to define the direction for our actions and, to achieve our goals. It is also vital to have public support and for individuals to take action towards a sustainable future.

